# DNMT1 and MBD2/3 Modulate Population Density-Dependent Plasticity in Terminal Oocyte Development in Bean Beetle, *Callosobruchus maculatus*

**DOI:** 10.3390/genes17060641

**Published:** 2026-05-31

**Authors:** Qianquan Chen, Yongqin Li, Yeying Wang

**Affiliations:** School of Life Sciences, Guizhou Normal University, Guiyang 550025, China; qqchen@gznu.edu.cn (Q.C.);

**Keywords:** phenotypic plasticity, RNAi, stored-product insect, insulin-like peptides, reproduction

## Abstract

**Background/Objectives**: The bean beetle (*Callosobruchus maculatus*) exhibits population density-dependent plasticity in the terminal oocyte maturation rate. *DNA methyltransferase 1* (*DNMT1*) plays a conserved function in reproduction that is independent of DNA methylation. However, whether *DNMT1* is involved in the population density-dependent reproductive plasticity of bean beetles remains unclear. **Methods**: Two and twenty pairs of beetles were reared with approximately 100 seeds per bottle to establish a low-density population and a high-density population, respectively. Quantitative real-time PCR was used to unveil the mRNA levels of *DNMT1*, *MBD2/3*, and *insulin-like peptides* (*ILPs*). RNA interference was used to determine the function of *DNMT1* and *MBD2/3* in terminal oocyte development. The length of terminal oocytes was measured under a microscope. **Results**: Individuals reared under high-population-density conditions showed a faster terminal oocyte maturation rate compared to those under low-density conditions. The bean beetle genome encodes *DNMT1* but lacks *DNMT3*, and only a single *methyl-DNA-binding domain protein* (*MBD2/3*) was identified. Population density could modulate the expression levels of both *DNMT1* and *MBD2/3*. RNA interference (RNAi)-mediated knockdown demonstrated that suppressing either *DNMT1* or *MBD2/3* significantly reduced the terminal oocyte maturation rate. Moreover, silencing *DNMT1* and *MBD2/3* resulted in decreased expression of *ILP3* and all *ILPs* in the fat body, respectively. *ILPs* are known to be involved in regulating terminal oocyte development. **Conclusions**: Taken together, these findings suggest that *DNMT1* and *MBD2/3* modulate the population density-dependent terminal oocyte maturation rate in the bean beetle by influencing the expression of *ILPs*.

## 1. Introduction

Phenotypic plasticity is a benefit for organisms to adapt to fluctuations in population density [1,2]. Consequently, many insects can sense population density and adjust various phenotypes, including behavior, body color, wing morphology, sensilla morphology, developmental rate, hormone metabolism, immunity, and reproduction [3]. For example, population density regulates the female sexual maturation rate in bean beetle (*C. maculatus*), a globally notorious stored-product pest. This species completes its life cycle on seeds from at least 35 plant species and rapidly adapts to novel hosts within a few generations [4]. It exhibits clear population density-dependent phenotypic plasticity in behavior, morphology, and reproduction [5]. Recent studies have implicated P450 genes and insulin-like peptides (ILPs) in regulating reproductive plasticity [6,7]. ILPs are key regulators of diverse physiological processes in insects, including metabolism, development, immunity, feeding behavior, stress resistance, diapause, lifespan, and reproduction [8]. They are expressed in the nervous system and various peripheral tissues, such as the midgut, imaginal discs, salivary glands, fat body, and ovary [8,9]. Four *ILP* genes have been identified in the bean beetle [7], and they regulate population density-dependent plasticity in the terminal oocyte maturity rate. Mechanistically, ILPs regulate vitellogenin (Vg) synthesis by modulating the phosphorylation status of forkhead box O (FOXO), which acts as a transcriptional repressor of Vg [10].

DNA methylation plays an important role in regulating gene expression in eukaryotes [11,12,13,14,15]. The predominant methylated nucleotide is 5-methylcytosine (5mC) [16], formed by DNA methyltransferases (DNMTs) via methyl group transfer from S-adenosyl methionine (SAM) to cytosine [17]. DNMT1 maintains existing methylation patterns, while DNMT3 mediates de novo methylation [18,19,20]. In mammals, methyl-DNA-binding domain proteins (MBDs, including MeCP2 and MBD1–6) recognize 5mC within CpG islands of promoter regions [21,22]. MBD2 and MBD3 share ~70% sequence identity; however, other MBDs show no significant similarity outside the conserved MBD domain [23,24]. MBDs bind methylated DNA via the methyl-DNA-binding domain [23]; notably, MBD3 lacks this domain and cannot bind DNA directly [25]. MBDs recruit histone deacetylases [26] and histone methyltransferases [27] to assemble a repressive nucleosomal array. Promoter DNA methylation strongly correlates with gene expression in plants and mammals [28,29,30]. Conversely, gene body 5mC is associated with enhanced transcription and regulates alternative splicing in cancer cells [31].

Similar to mammals, some insects, such as the honey bee (*Apis mellifera*), encode both *DNMT1* and *DNMT3*. However, others, including the silkworm (*Bombyx mori*) and the red flour beetle (*Tribolium castaneum*), encode only *DNMT1*, whereas species such as the fruit fly (*Drosophila melanogaster*) lack both genes [32]. *DNMT1* plays important roles in insect reproduction. For example, in the large milkweed bug (*Oncopeltus fasciatus*), *DNMT1* is involved in physiological processes such as egg laying and embryo development [33]. In the silkworm, RNA interference (RNAi) targeting *DNMT1* reduces egg hatch rates [34]. Similarly, *DNMT1* is critical for oogenesis and embryogenesis in the red flour beetle [35] and modulates the female reproductive response to temperature in the bean beetle [36]. In most insects, 5mC sites are found within the gene bodies of highly conserved, highly expressed, single-copy genes [37,38], occurring predominantly at CG dinucleotides. For instance, in the silkworm, 83.61%, 13.47%, and 2.93% of 5mC sites are located in CG, CHG, and CHH contexts, respectively [39]. Despite the presence of 5mC, its functional link to gene expression remains unclear. In the milkweed bug, *DNMT1* knockdown leads to significant alterations in DNA methylation patterns but decreases mRNA levels for only 1% of methylated genes, indicating that 5mC may not directly regulate gene expression [33]. Similarly, in the whitefly (*Bemisia tabaci*), genomic DNA from both the dsGFP- and dsDNMT1-treated groups contained approximately 5% CpG methylation [40], and in the silkworm, expression levels of most methylated genes remain unchanged after demethylation [39]. Across a range of insects, including beetles, ants, and bees, DNA methylation shows no clear association with gene expression [41]. Moreover, recent whole-genome analyses have revealed no consistent relationship between DNA methylation and alternative splicing in these insects [41]. Together, these findings suggest that *DNMT1* has a conserved function in reproduction that is independent of its role in DNA methylation [41]. Insects typically possess only one MBD, often referred to as MBD2/3 due to its high homology to vertebrate MBD2 and MBD3 [22].

The bean beetle exhibits clear population density-dependent phenotypic plasticity in reproduction; meanwhile, *DNMT1* has a conserved function in reproduction. However, the role of *DNMT1* in mediating the population-dependent reproductive plasticity in the bean beetle remains unexplored. Whether MBD2/3 shares a similar gene regulatory function with *DNMT1* remains unknown.

## 2. Materials and Methods

### 2.1. Insects

Bean beetles were maintained in the School of Life Sciences, at Guizhou Normal University [5,6,7]. They were reared with mung beans in tissue culture bottles (0.2 L). Two pairs of beetles were reared with approximately 100 seeds per bottle to establish a low-density population; 20 pairs of beetles were reared with approximately 100 seeds per bottle to establish a high-density population. These two populations were reared for ten generations. Our previous study indicated that under the high beetle density, the mean eclosion efficiency, i.e., the number of holes per seed, was 4.2 times greater than that in the low beetle density [5]. All beetles were reared at 28 ± 2 °C with a 14:10 light:dark photoperiod and 50–70% humidity.

### 2.2. Measurement of Terminal Oocyte Length

Female beetles reach full maturity approximately 30 h after adult emergence; therefore, females were collected for RNA extraction and terminal oocyte measurement at about 12 h after adult emergence [6,7]. Some females were immediately placed in liquid nitrogen and subsequently stored in a −80 °C freezer for RNA extraction. The remaining females were fixed in 4% paraformaldehyde solution for three days. The ovaries were dissected into individual ovarioles. Images of terminal oocytes, located at the tip of each ovariole, were captured using a microscope (SZX7; Olympus, Tokyo, Japan). The length of the terminal oocytes was measured with the cellSensEntry software (Olympus, Tokyo, Japan). Eight terminal oocytes from the same female were measured, and their lengths were averaged. Fifteen females (biological replications) were used for each treatment (high-density population vs. low-density population; dsGFP vs. dsDNMT1; dsGFP vs. dsMBD2/3).

### 2.3. Quantitative Real-Time PCR

Female beetles were dissected into brain, fat body, and ovary. Brains from 12 individuals were pooled together as one biological replicate, and six biological replicates were analyzed for each group (high population density vs. low population density) or each treatment (dsGFP vs. dsDNMT1; dsGFP vs. dsMBD2/3). Similarly, fat bodies or ovaries from 12 individuals were pooled together as one biological replicate, and six biological replicates were analyzed for each group or each treatment. Total RNA was extracted using TriQuick Reagent (Solarbio, Beijing, China). The RNA concentration and quality were measured with a Biotek Epoch 2 (Agilent Technologies, Santa Clara, USA). Two micrograms of total RNA was used for cDNA synthesis with FastKing gDNA Dispelling RT Supermix (Tiangen, Beijing, China). The mRNA levels were determined using Talent qPCR PreMix (SYBR Green) (Tiangen, Beijing, China) and normalized to the expression of ribosome protein 49 (rp49). PCR amplification was performed on a Thermo QuantStudio 3 (Thermo Fisher Scientific Pte Ltd., Marsiling, Singapore). Melting curve analysis was used to confirm amplification specificity. Relative mRNA levels were calculated with the 2^−ΔΔCt^ method. Primer sequences are listed in Table S1.

### 2.4. RNA Interference

A partial fragment of *DNMT1* was amplified from cDNA with gene-specific primers (Table S1). The PCR product was inserted into the PGEM-T Easy vector (Promega, Madison, WI, USA). The T7 promoter sequence was added to the 5′ end of the forward primer (T7-F) and reverse primer (T7-R). One double-stranded RNA (dsRNA) synthesis template was amplified from the vector containing the *DNMT1* sequence using the combination of T7-F and R, and the other template was amplified using the combination of F and T7-R. DsRNAs were generated with the T7 RiboMAX^TM^ Express Large Scale RNA Production System (Promega, Madison, WI, USA). DsRNAs targeting MBD2/3 were synthesized following the same protocol. DsRNA targeting green fluorescent protein (dsGFP) was used as a control. The lengths of dsGFP, dsDNMT1, and dsMBD2/3 were 420, 549, and 382 bp, respectively (Table S1). High-density population individuals were used for injection. At the pupal stage (approximately three days before adult emergence), approximately 1 µg (0.3 µL) of dsRNA was injected into each beetle through the abdomen using a nanoliter injector 2000 (World Precision Instruments, Sarasota, FL, USA) with a microglass needle. Female beetles were collected for silencing efficiency analysis and measurement of terminal oocytes at about 12 h after adult emergence.

### 2.5. Statistical Analysis

Data normality was assessed using the Shapiro–Wilk test, and homogeneity of variances was assessed using the Bartlett test. For normally distributed data with equal variances, *t*-test and one-way ANOVA were used for two-group and three-group comparisons, respectively. For data that did not meet the assumptions of normality and/or equal variance, the Wilcoxon rank sum exact test and Kruskal–Wallis test were used for two-group and three-group comparisons, respectively. Statistical analyses were conducted using R version 4.5.0. Differences were considered significant if *p* < 0.05. Data are presented as the mean ± standard error of the mean (SEM).

## 3. Results

### 3.1. Terminal Oocyte Maturation Rate

**Figure 1:**
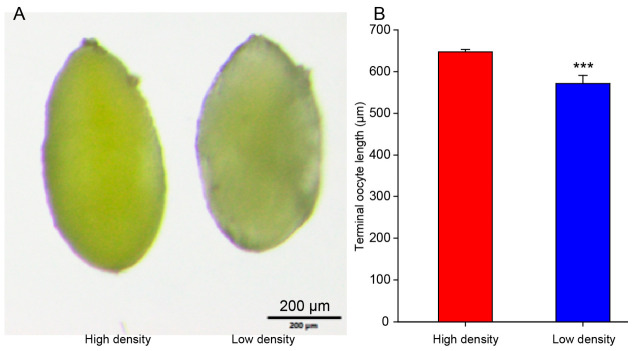
Population density modulates terminal oocyte maturation rate. (**A**) Terminal oocyte morphology. (**B**) The length of terminal oocytes. Data are presented as mean ± standard error of the mean (SEM). *n* = 15 for each group, Wilcoxon rank sum test, ***, *p* < 0.0001.

The length of terminal oocytes is usually used as an indicator of the female sexual maturation rate. Female bean beetles reach full maturity at approximately 30 h after adult emergence; therefore, females were collected for terminal oocyte measurement at about 12 h after adult emergence [6,7]. The terminal oocyte length of high-population-density individuals (H) was longer than that of low-population-density individuals (L) (*n* = 15 for each group, Wilcoxon rank sum test, *p* < 0.0001) (Figure 1).

### 3.2. Expression of DNMT1 and MBD2/3

**Figure 2:**
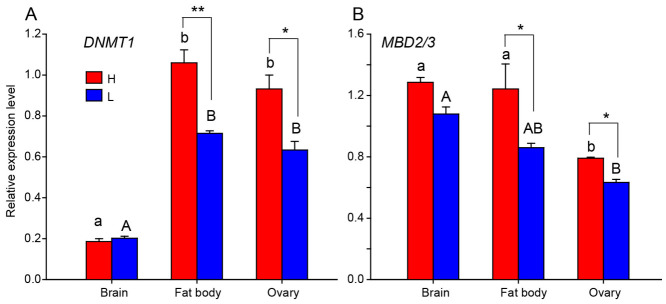
Tissue-specific expression of *DNMT1* and *MBD2/3* under different population densities. (**A**) *DNMT1* mRNA levels. For high-population-density bean beetles (H), *n* = 6 for each tissue, ANOVA, *p* < 0.001; for low-population-density bean beetles (L), *n* = 6 for each tissue, ANOVA, *p* < 0.001. Entries labeled with different letters indicate significantly different means, and those labeled with similar letters indicate non-significantly different means. Lowercase letters and uppercase letters are used for H and L, respectively. Brain, *n* = 6 for each group, *t*-test, *p* = 0.78; fat body, *n* = 6 for each group, *t*-test, *p* = 0.002; ovary, *n* = 6 for each group, *t*-test, *p* = 0.05. *, *p* < 0.05; **, *p* < 0.01. (**B**) *MBD2/3* mRNA levels. H, *n* = 6 for each tissue, Kruskal–Wallis test, *p* = 0.011; L, *n* = 6 for each tissue, ANOVA, *p* < 0.001. Brain, *n* = 6 for each group, *t*-test, *p* = 0.071; fat body, *n* = 6 for each group, *t*-test, *p* = 0.048; ovary, *n* = 6 for each group, *t*-test, *p* = 0.046.

*DNMT1* was identified from genomic and transcriptomic data of the bean beetle, while *DNMT3* was not detected. The genome encoded a single *MBD*, *MBD2/3*. The expression level of *DNMT1* was significantly higher in the fat body and ovary than that in the brain (H, *n* = 6 for each tissue, ANOVA, *p* < 0.001; L, *n* = 6 for each tissue, ANOVA, *p* < 0.001) (Figure 2A). *DNMT1* expression in the fat body and ovary was higher in high-population-density beetles compared to low-population-density individuals (fat body, *n* = 6 for each group, *t*-test, *p* = 0.002; ovary, *n* = 6 for each group, *t*-test, *p* = 0.05). Population density did not significantly affect *DNMT1* expression in the brain (*n* = 6 for each group, *t*-test, *p* = 0.78). In contrast, the expression level of *MBD2/3* was higher in the brain and fat body than that in the ovary (H, *n* = 6 for each tissue, Kruskal–Wallis test, *p* = 0.011; L, *n* = 6 for each tissue, ANOVA, *p* < 0.001) (Figure 2B). *MBD2/3* was higher in the fat body and ovary of individuals reared under high-population-density conditions (fat body, *n* = 6 for each group, *t*-test, *p* = 0.048; ovary, *n* = 6 for each group, *t*-test, *p* = 0.046), while its expression level in the brain remained unaffected by population density (*n* = 6 for each group, *t*-test, *p* = 0.071). Taken together, these results demonstrated that population density was associated with altered expression of *DNMT1* and *MBD2/3* in the fat body and ovary.

### 3.3. Functional Analysis of DNMT1 and MBD2/3 in Terminal Oocyte Development

**Figure 3:**
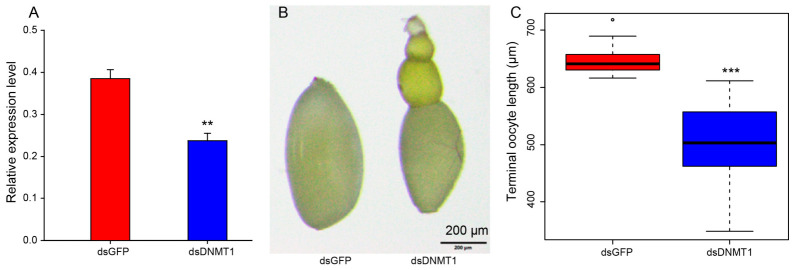
Effect of silencing *DNMT1* on terminal oocyte development. (**A**) Silencing efficiency of *DNMT1* in fat body (*n* = 6 for each treatment, *t*-test, *p* < 0.001). (**B**) Terminal oocyte morphology following *DNMT1* knockdown. (**C**) The length of terminal oocytes following *DNMT1* knockdown (*n* = 15 for each treatment, Wilcoxon rank sum exact test, *p* < 0.0001). **, *p* < 0.01; ***, *p* < 0.0001.

**Figure 4:**
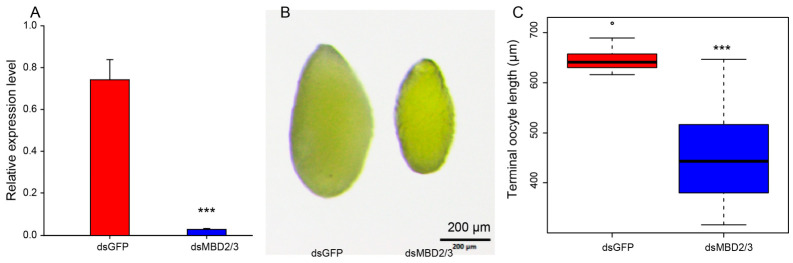
Effect of silencing *MBD2/3* on terminal oocyte development. (**A**) Silencing efficiency of *MBD2/3* in fat body (*n* = 6 for each treatment, Wilcoxon rank sum exact test, *p* = 0.0022). (**B**) Terminal oocyte morphology following *MBD2/3* knockdown. (**C**) The length of terminal oocytes following *MBD2/3* knockdown (*n* = 15 for each treatment, Wilcoxon rank sum exact test, *p* < 0.0001). ***, *p* < 0.0001.

Due to the upregulation of *DNMT1* and *MBD2/3* in high-density individuals, we reduced their expression in high-density individuals by RNAi to unveil their functions in terminal oocyte development. Double-stranded RNAs targeting *DNMT1* (dsDNMT1) and *MBD2/3* (dsMBD2/3) were designed. Following dsDNMT1 injection, *DNMT1* mRNA levels in the fat body decreased by 38% (Figure 3A) (*n* = 6 for each treatment, *t*-test, *p* < 0.001), resulting in a 23% decrease in the length of terminal oocytes (Figure 3B,C) (*n* = 15 for each treatment, Wilcoxon rank sum exact test, *p* < 0.0001). After dsMBD2/3 injection, *MBD2/3* mRNA levels in the fat body were reduced by 95% (Figure 4A) (*n* = 6 for each treatment, Wilcoxon rank sum exact test, *p* = 0.0022), accompanied by a 31% reduction in the length of terminal oocytes (Figure 4B,C) (*n* = 15 for each treatment, Wilcoxon rank sum exact test, *p* < 0.0001). Taken together, both *DNMT1* and *MBD2/3* were involved in regulating population density-dependent terminal oocyte development.

### 3.4. Regulation of ILP Expression by DNMT1 and MBD2/3

**Figure 5:**
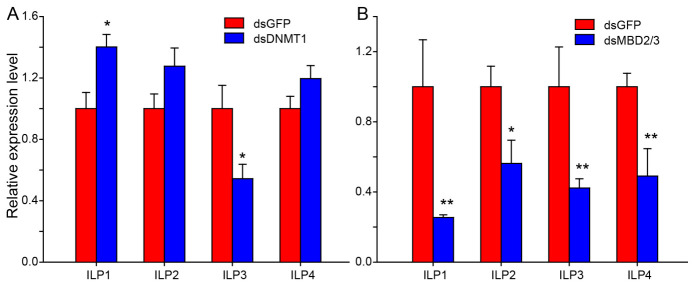
Expression of *insulin-like peptides* (*ILPs*) following knockdown of *DNMT1* or *MBD2/3*. (**A**) mRNA levels of *ILPs* after silencing *DNMT1* (*ILP1*, *n* = 5 for each treatment, *t*-test, *p* = 0.018; *ILP2*, *n* = 5 for each treatment, *t*-test, *p* = 0.11; *ILP3*, *n* = 6 for each treatment, *t*-test, *p* = 0.032; *ILP4*, *n* = 6 for each treatment, *t*-test, *p* = 0.13). (**B**) mRNA levels of *ILPs* after silencing *MBD2/3* (*ILP1*, *n* = 5 for each treatment, Wilcoxon rank sum exact test, *p* = 0.008; *ILP2*, *n* = 6 for each treatment, *t*-test, *p* = 0.033; *ILP3*, *n* = 6 for each treatment, Wilcoxon rank sum exact test, *p* = 0.009; *ILP4*, *n* = 6 for each treatment, *t*-test, *p* = 0.009). *, *p* < 0.05; **, *p* < 0.01.

Given the established role of *ILPs* in terminal oocyte development, we quantified the expression of *ILPs* in the fat body following knockdown of *DNMT1* and *MBD2/3*. The mRNA level of *ILP1* increased by 1.4-fold after dsDNMT1 injection (Figure 5A, *n* = 5 for each treatment, *t*-test, *p* = 0.018). However, the mRNA level of *ILP3* was reduced by 46% (Figure 5A, *n* = 6 for each treatment, *t*-test, *p* = 0.032). Knockdown of *DNMT1* had no significant effect on *ILP2* and *ILP4* expression (*ILP2*, *n* = 5 for each treatment, *t*-test, *p* = 0.11; *ILP4*, *n* = 6 for each treatment, *t*-test, *p* = 0.13). The mRNA levels of *ILP1*, *ILP2*, *ILP3*, and *ILP4* decreased by 74.59%, 43.73%, 57.74%, and 50.96% after dsMBD2/3 injection, respectively (Figure 5B, *ILP1*, *n* = 5 for each treatment, Wilcoxon rank sum exact test, *p* = 0.008; *ILP2*, *n* = 6 for each treatment, *t*-test, *p* = 0.033; *ILP3*, *n* = 6 for each treatment, Wilcoxon rank sum exact test, *p* = 0.009; *ILP4*, *n* = 6 for each treatment, *t*-test, *p* = 0.009). Taken together, both *DNMT1* and *MBD2/3* could affect the expression of *ILP3*.

## 4. Discussion

Bean beetles displayed population density-dependent phenotypic plasticity in reproduction. Female beetles lay eggs on the surface of seeds; then, the eggs hatch as larvae, which burrow into the seeds for further development [5]. Under low population density, abundant seeds are available for oviposition, leading females to lay more eggs to increase offspring numbers. With the increase in population density, females have to compete for seeds on which to lay eggs. Under high population density, females with a faster terminal oocyte maturation rate can oviposit earlier, and earlier-hatching larvae may exhibit earlier development. Similarly, population density regulates the female sexual maturity rate and the offspring developmental rate in locusts [42,43]. The intensity of cannibalism increases with population density [44], and individuals that develop earlier may have an advantage in killing and consuming conspecifics. In summary, insects can adjust their terminal oocyte maturation rate to enhance offspring fitness.

In the present study, *DNMT1* expression was significantly higher in the fat body and ovary than in the brain, whereas *MBD2/3* showed elevated expression in the brain and fat body relative to the ovary. Both genes were upregulated in the fat body and ovary under high population density, indicating that population density could modulate the expression of *DNMT1* and *MBD2/3*. RNAi targeting *DNMT1* (dsDNMT1) reduced its expression level in the fat body by 38%, while dsMBD2/3 achieved 95% knockdown, reflecting gene-specific RNAi efficiencies as documented elsewhere [7]. Consequently, the lengths of terminal oocytes decreased by 23% and 31% after dsDNMT1 and dsMBD2/3 injection, respectively. *DNMT1* and *MBD2/3* function biologically as proteins; however, due to the lack of antibodies against DNMT1 and MBD2/3 in *C. maculatus*, we did not detect their protein levels. Factors such as mRNA stability, large transcripts, and the accessibility of the region on the target gene can resist degradation, attenuating the knockdown efficiency. The transcripts of *DNMT1* and *MBD2/3* were 4755 and 1112 nt, respectively. The large size of *DNMT1* might contribute to its low knockdown efficiency. In RNA interference experiments, off-target effects are a notorious confounding variable; however, our current study does not include a second non-overlapping fragment, and we have explicitly acknowledged this as a limitation. *DNMT1* is known to be involved in reproduction and development. For example, it regulates egg laying and embryo development in the large milkweed bug [33]. Its expression level influences egg hatch rates in the silkworm [34]. *DNMT1* plays a critical role in oogenesis and embryogenesis in the red flour beetle [35]. It is also involved in modulating the female reproductive response to temperature in the bean beetle [36]. In the milkweed bug, knockdown of *DNMT1* leads to significant alterations in DNA methylation patterns; however, only 1% of methylated genes show decreases in mRNA levels, indicating that 5mC may not directly regulate gene expression [33]. In whitefly, genomic DNA from dsGFP- and dsDNMT1-treated females contains approximately 5% CpG methylation [40]. In the silkworm, the expression levels of most methylated genes do not change after demethylation [39]. Furthermore, DNA methylation shows no clear association with gene expression and alternative splicing across a range of insects, including beetles, ants, and bees [41]. Collectively, these findings suggest that *DNMT1* has a conserved function in reproduction that is independent of DNA methylation [41]. However, we did not explore the effect of population density and dsDNMT1 on the level of 5mC.

After dsDNMT1 injection, the mRNA levels of *ILP1* and *ILP3* increased by 1.4-fold and decreased by 46%, respectively, indicating that *DNMT1* had an opposite effect on the expression of *ILP1* and *ILP3*. After dsMBD2/3 injection, the expression levels of all four *ILPs* were reduced. ILPs are involved in metabolism, development, immunity, feeding behavior, stress resistance, diapause, lifespan, and reproduction [8]. They are expressed not only in the nervous system but also in various peripheral tissues, including the midgut, imaginal discs, salivary glands, fat body, and ovaries [8,9]. ILPs can regulate vitellogenin (Vg) synthesis in the fat body by modulating the phosphorylation status of forkhead box O (FOXO), which acts as a transcriptional repressor of Vg [10]. The fat body modulates energy storage and release, synthesizes hemolymph proteins, and produces vitellogenin [45]. Vitellogenin is processed into yolk protein for terminal oocyte uptake. Four *ILPs* have been identified in the bean beetle [7], and they can regulate the terminal oocyte maturation rate. Among these four *ILPs*, *ILP3* exhibits compensatory upregulation when *ILP1* or *ILP2* is suppressed [7]. Although both *DNMT1* and *MBD2/3* can affect the expression of *ILP3*, the underlying molecular mechanism requires further experiments such as methylation profiling, chromatin assays and promoter analysis.

## 5. Conclusions

Population density modulates the terminal oocyte maturation rate as well as the expression of *DNMT1* and *MBD2/3* in bean beetles. The RNA interference results show that both *DNMT1* and *MBD2/3* regulate the terminal oocyte maturation rate. *DNMT1* affects the expression of *ILP1* and *ILP3*; however, *MBD2/3* affects the expression of all *ILPs*. Together, *DNMT1* and *MBD2/3* modulate population density-dependent oocyte development by influencing the expression of *ILPs*.

## Data Availability

The data presented in this study are openly available in [Baidu Cloud] at [raw data for DNMT1 and MBD23 modulate population density-dependent plasticity in terminal oocyte development in bean beetle], reference number [kns5], [https://pan.baidu.com/s/1bClGxWkPARK-V4lEKHBXuA] [kns5] (accessed on 13 May 2026).

## Supplementary Materials

The following supporting information can be downloaded at: https://www.mdpi.com/article/10.3390/genes17060641/s1, Table S1: Primers used for RT-PCR and RNAi.

## References

[1] Brass D.P., Cobbold C.A., Ewing D.A., Purse B.V., Callaghan A., White S.M. (2021). Phenotypic plasticity as a cause and consequence of population dynamics. Ecol. Lett..

[2] Yang D., Jin Y., He X., Dong A., Wang J., Wu R. (2021). Inferring multilayer interactome networks shaping phenotypic plasticity and evolution. Nat. Commun..

[3] Barnes A.I., Siva-Jothy M.T. (2000). Density-dependent prophylaxis in the mealworm beetle *Tenebrio molitor* L. (Coleoptera: Tenebrionidae): Cuticular melanization is an indicator of investment in immunity. Proc. Biol. Sci..

[4] Arnqvist C.F.R. (2007). Rapid adaptation to a novel host in a seed beetle (*Callosobruchus maculatus*): The role of sexual selection. Evol. Int. J. Org. Evol..

[5] Chen Q.Q., Ma J.J., Yang H., Gong J.H., Gong X.Q., Weng Q.B. (2019). Seed-coat colour affects oviposition in the bean beetle, *Callosobruchus maculatus* (Coleoptera: Chrysomelidae). Ann. Zool. Fenn..

[6] Chen Q.Q., Li Y.Q., Fang Z., Wu Q.S., Tan L.T., Weng Q.B. (2024). CYP4BN4v7 regulates the population density dependent oocyte maturity rate in bean beetles. Sci. Rep..

[7] Li Y.Q., Fang Z., Tan L.T., Wu Q.S., Liu Q.P., Wang Y.Y., Weng Q.B., Chen Q.Q. (2024). Gene redundancy and gene compensation of insulin-like peptides in the oocyte development of bean beetle. PLoS ONE.

[8] Semaniuk U., Strilbytska O., Malinovska K., Storey K.B., Vaiserman A., Lushchak V., Lushchak O. (2021). Factors that regulate expression patterns of insulin-like peptides and their association with physiological and metabolic traits in *Drosophila*. Insect Biochem. Mol. Biol..

[9] Chowański S., Walkowiak-Nowicka K., Winkiel M., Marciniak P., Urbański A., Pacholska-Bogalska J. (2021). Insulin-like peptides and cross-talk with other factors in the regulation of insect metabolism. Front. Physiol..

[10] Roy S., Saha T.T., Zou Z., Raikhel A.S. (2018). Regulatory pathways controlling female insect reproduction. Annu. Rev. Entomol..

[11] Huang W.Y., Hsu S.D., Huang H.Y., Sun Y.M., Chou C.H., Weng S.L., Huang H.D. (2015). MethHC: A database of DNA methylation and gene expression in human cancer. Nucleic Acids Res..

[12] Hong J., Rhee J.K. (2022). Genomic Effect of DNA methylation on gene expression in colorectal cancer. Biology.

[13] Hwang J.H., An S.M., Kwon S., Park D.H., Kim T.W., Kang D.G., Yu G.E., Kim I.S., Park H.C., Ha J. (2017). DNA methylation patterns and gene expression associated with litter size in Berkshire pig placenta. PLoS ONE.

[14] Maghbooli Z., Hossein-Nezhad A., Adabi E., Asadollah-Pour E., Sadeghi M., Mohammad-Nabi S., Rad L.Z., Hosseini A.A.M., Radmehr M., Faghihi F. (2018). Air pollution during pregnancy and placental adaptation in the levels of global DNA methylation. PLoS ONE.

[15] Wang J., Cui J.H., Chen R., Deng Y.C., Liao X., Wei Y.L., Li X.H., Su M., Yu J.H., Yi P. (2017). Prenatal exposure to lipopolysaccharide alters renal DNA methyltransferase expression in rat offspring. PLoS ONE.

[16] Mattei A.L., Bailly N., Meissner A. (2022). DNA methylation: A historical perspective. Trends Genet..

[17] Zhang N.F. (2018). Role of methionine on epigenetic modification of DNA methylation and gene expression in animals. Anim. Nutr..

[18] Lyko F. (2018). The DNA methyltransferase family: A versatile toolkit for epigenetic regulation. Nat. Rev. Genet..

[19] Laisné M., Gupta N., Kirsh O., Pradhan S., Defossez P.A. (2018). Mechanisms of DNA methyltransferase recruitment in mammals. Genes.

[20] Loaeza-Loaeza J., Beltran A.S., Hernandez-Sotelo D. (2020). DNMTs and impact of CpG content, transcription factors, consensus motifs, lncrnas, and histone marks on DNA methylation. Genes.

[21] Gigek C.O., Chen E.S., Smith M.A. (2016). Methyl-CpG-binding protein (MBD) family: Epigenomic read-outs functions and roles in tumorigenesis and psychiatric diseases. J. Cell. Biochem..

[22] Hendrich B., Tweedie S. (2003). The methyl-CpG binding domain and the evolving role of DNA methylation in animals. Trends Genet..

[23] Fatemi M., Wade P.A. (2006). MBD family proteins: Reading the epigenetic code. J. Cell Sci..

[24] Song X.W., Zhang Y.M., Zhong Q.S., Zhan K.M., Bi J.X., Tang J., Xie J., Li B. (2020). Identification and functional characterization of methyl-CpG binding domain protein from *Tribolium castaneum*. Genomics.

[25] Baubec T., Ivánek R., Lienert F., Schübeler D. (2013). Methylation-dependent and -independent genomic targeting principles of the MBD protein family. Cell.

[26] Dobosy J.R., Selker E.U. (2001). Emerging connections between DNA methylation and histone acetylation. Cell. Mol. Life Sci..

[27] Fuks F., Hurd P.J., Wolf D., Nan X., Bird A.P., Kouzarides T. (2003). The Methyl-CpG-binding protein MeCP2 links DNA methylation to histone methylation. J. Biol. Chem..

[28] Bogan S.N., Yi S. (2024). Potential role of DNA methylation as a driver of plastic responses to the environment across cells, organisms, and populations. Genome Biol. Evol..

[29] Huang H.Q., Guo C.Y., Cheng S.P., Wang Z. (2026). DNA methylation dynamics in plant abiotic stress response: Mechanisms, memory, and breeding applications. Genes.

[30] Serej O., Kowalik M.K., Rekawiecki R. (2026). DNA methylation in the ovary and uterus of mammalian animal models: Implications for reproductive function. Genes.

[31] Yang X., Han H., De Carvalho D.D., Lay F.D., Jones P.A., Liang G. (2014). Gene body methylation can alter gene expression and is a therapeutic target in cancer. Cancer Cell.

[32] Bewick A.J., Vogel K.J., Moore A.J., Schmitz R.J. (2017). Evolution of DNA methylation across Insects. Mol. Biol. Evol..

[33] Bewick A.J., Sanchez Z., McKinney E.C., Moore A.J., Moore P.J., Schmitz R.J. (2019). DNMT1 is essential for egg production and embryo viability in the large milkweed bug, *Oncopeltus fasciatus*. Epigenetics Chromatin.

[34] Xiang H., Li X., Dai F., Xu X., Tan A., Chen L., Zhang G., Ding Y., Li Q., Lian J. (2013). Comparative methylomics between domesticated and wild silkworms implies possible epigenetic influences on silkworm domestication. BMC Genom..

[35] Schulz N.K.E., Wagner C.I., Ebeling J., Raddatz G., Diddens-de Buhr M.F., Lyko F., Kurtz J. (2018). DNMT1 has an essential function despite the absence of CpG DNA methylation in the red flour beetle Tribolium castaneum. Sci. Rep..

[36] McCaw B.A., Leonard A.M., Stevenson T.J., Lancaster L.T. (2024). A role of epigenetic mechanisms in regulating female reproductive responses to temperature in a pest beetle. Insect Mol. Biol..

[37] Glastad K.M., Hunt B.G., Goodisman M.A.D. (2014). Evolutionary insights into DNA methylation in insects. Curr. Opin. Insect Sci..

[38] Hunt B.G., Glastad K.M., Yi S.V., Goodisman M.A. (2013). The function of intragenic DNA methylation: Insights from insect epigenomes. Integr. Comp. Biol..

[39] Xu G.F., Lyu H., Yi Y.Q., Peng Y.L., Feng Q.L., Song Q.S., Gong C.C., Peng X.Z., Palli S.R., Zheng S.C. (2021). Intragenic DNA methylation regulates insect gene expression and reproduction through the MBD/Tip60 complex. Iscience.

[40] Shelby E.A., McKinney E.C., Cunningham C.B., Simmons A.M., Moore A.J., Moore P.J. (2023). The role of DNMT1 in oocyte development. J. Insect Physiol..

[41] Duncan E.J., Cunningham C.B., Dearden P.K. (2022). Phenotypic plasticity: What has DNA methylation got to do with it?. Insects.

[42] Chen Q., He J., Ma C., Yu D., Kang L. (2015). Syntaxin 1A modulates the sexual maturity rate and progeny egg size related to phase changes in locusts. Insect Biochem. Mol. Biol..

[43] He J., Chen Q., Wei Y., Jiang F., Yang M., Hao S., Guo X., Chen D., Kang L. (2016). MicroRNA-276 promotes egg-hatching synchrony by up-regulating *brm* in locusts. Proc. Natl. Acad. Sci. USA.

[44] Chang H., Cassau S., Krieger J., Guo X., Knaden M., Kang L., Hansson B.S. (2023). A chemical defense deters cannibalism in migratory locusts. Science.

[45] Arrese E.L., Soulages J.L. (2010). Insect Fat body: Energy, metabolism, and regulation. Annu. Rev. Entomol..

