# Supplementary material for: DNMT1 and MBD2/3 Modulate Population Density-Dependent Plasticity in Terminal Oocyte Development in Bean Beetle, Callosobruchus maculatus
Source: Genes (Basel). 2026 May 31;17(6):641. doi: 10.3390/genes17060641 (PMC13299485; doi:10.3390/genes17060641)
Supplement: Supplementary file 1 [file genes-17-00641-s001.zip › genes-4345280-supplementary.pdf]

Table S1. Primers used for RT-PCR and RNAi.

Primer name	Gene ID	Location	Exon	Sequence (5' to 3')
q-rp49 F	GEUD01120135.1	197-216	✓	GCAACTGGCGTAAACCTAAA
q-rp49 R		319-338	✓	TGTACTAGCACCTTCCTGAA
q-DNMT1 F	GEUD01136775.1	1696-1677	✓	CGGCGACTTCGTAATGCTGA
q-DNMT1 R		1809-1828	✓	CAGATGTCTCACCGAGGATG
q-MBD2/3 F	GEUD01176596.1	151-170	✓	ATGGCGAGCATAACAATCGA
q-MBD2/3 R		320-339	✓	CTTGAAGATGCTGGCTGTCT
q-ILP1 F	GEUD01205412.1	160-179	✓	TGAAACAGCGTTACAGTCCG
q-ILP1 R		320-339	✓	GGTTGGCTTCTTCTGGTTAG
q-ILP2 F	GEUD01073347.1	357-376	✓	AGCAGGTACTCAAGACATCG
q-ILP2 R		536-555	✓	CTCAGCATGGAAGCGTCAGG
q-ILP3 F	GEUD01082331.1	255-274	✓	CTGACTCTAAACACGGCACT
q-ILP3 R		361-380-	✓	GAGACAGAACTCGGCCAAAA
q-ILP4 F	GEUD01001279.1	102-118	✓	CATCACCATACATGCACCAT
q-ILP4 R		245-264	✓	GATAACTCGTCGTCATCCTT
RNAi-GFP F	MT612434.1	2322-2340	✓	CACAAGTTCAGCGTGTCCG
RNAi-GFP R		2723-2741	✓	GTTCACCTTGATGCCGTTC
RNAi-DNMT1 F	GEUD01136775.1	2967-2986	✓	TGAAATGTTAGTTGGAGGAC
RNAi-DNMT1 R		3496-3515	✓	AACATACGCTGAAAGTGAGA
RNAi-MBD2/3 F	GEUD01176596.1	288-307	✓	AAGTGATGGTTGTTTGGTGC
RNAi-MBD2/3 R		651-670	✓	CAGCATGAACCAATGGCTGT
T7-RNAi-GFP F				TAATACGACTCACTATAGGCACAAGTTCAGCGTGTCCG
T7-RNAi-GFP R				TAATACGACTCACTATAGGGTTCACCTTGATGCCGTTC
T7-RNAi-DNMT1 F				TAATACGACTCACTATAGGTGAAATGTTAGTTGGAGGAC
T7-RNAi-DNMT1 R				TAATACGACTCACTATAGGAACATACGCTGAAAGTGAGA
T7-RNAi-MBD2/3 F				TAATACGACTCACTATAGGAAGTGATGGTTGTTTGGTGC
T7-RNAi-MBD2/3 R				TAATACGACTCACTATAGGCAGCATGAACCAATGGCTGT
